# West Siberian Soil Resistome: Mobile Antibiotic Resistance in Agricultural Microbiomes

**DOI:** 10.3390/antibiotics15050502

**Published:** 2026-05-17

**Authors:** Anna Evgenevna Skotareva, Ekaterina Alexeevna Sokolova, Elena Nikolaevna Voronina

**Affiliations:** 1 Institute of Chemical Biology and Fundamental Medicine, Siberian Branch of the Russian Academy of Sciences, 630090 Novosibirsk, Russia; skotareva04@mail.ru (A.E.S.); sokolova_ea@1bio.ru (E.A.S.); 2 Department of Molecular Biology and Biotechnology, Faculty of Natural Sciences, Novosibirsk State University, 630090 Novosibirsk, Russia

**Keywords:** antibiotic resistance, soil microbiome, mobile genetic elements, metagenomics, horizontal gene transfer, integrons, agroecosystems, West Siberia

## Abstract

**Background/Objectives:** Soil microbiomes in agroecosystems are natural reservoirs of antibiotic resistance genes (ARGs) and mobile genetic elements (MGEs), creating conditions for horizontal gene transfer (HGT) to clinically relevant bacteria. Southern West Siberia—a globally significant grain-producing region—lacks metagenomic characterization of its soil resistome. This study aimed to establish the first baseline profile of resistome and mobilome composition for West Siberian agricultural soils. **Methods:** Twelve composite soil samples were collected from agroecosystems under seven crop types across diverse soil types in southern West Siberia (September 2022). Shotgun metagenomics was performed on an Illumina NovaSeq 6000 platform. Taxonomic profiling used Kraken2/Bracken; ARG annotation used Prokka/DeepARG (identity ≥ 70%, probability score ≥ 0.8); while MGE characterization used Platon, HMMER v3.3.2, and Prokka-based integrase annotation. Resistome load was normalized to the single-copy housekeeping gene *rpoB*; ARG–MGE associations were defined as co-localization within 10 kb on the same contig. **Results:** Microbial communities were dominated by *Pseudomonadota* and *Bacillota*, with a stable core of *Streptomycetaceae*, *Nitrobacteraceae*, and *Sphingomonadaceae*. Normalized resistome load (N/*rpoB* 2.30–5.37) indicated moderate anthropogenic pressure. Dominant ARGs included efflux pumps (*emrA*, *drrA*, *tetA*, *bcr*, *fsr*), target modification (*lnrL*), and lipid A modification (*arnA*) genes. Class 1 integron integrase (*intI1*/*rpoB* 0.64–1.59) was detected in all 12 samples, exceeding unity in 9 of 12. ARG–MGE co-localizations were found in 11 of 12 samples. In sample Mg_155, genes *emrA–emrB* and *bcr* (NODE_16) and *arnA* and *lnrL* (NODE_6) were each independently associated with distinct prophage *IntA* integrase copies within *Pseudomonas* contigs, documenting multiple parallel horizontal transfer events encompassing resistance to five antibiotic classes. **Conclusions:** This work establishes the first metagenomic baseline of resistome and mobilome for West Siberian agroecosystems. The obtained data indicate moderate anthropogenic pressure on soil microbiomes, consistent with temperate agricultural systems with limited organic fertilizer input. The detected ARG–MGE co-localizations and evidence of prophage-mediated transfer of resistance determinants beyond their natural hosts suggest that mobilization potential in the region warrants consideration in future AMR monitoring programs.

## 1. Introduction

The properties of soil ecosystems are largely determined by the composition and functional activity of the microbial communities inhabiting them. Soil microbiomes participate in key biogeochemical cycles, organic matter transformation, and soil fertility formation. Moreover, they serve as natural reservoirs of genetic determinants of antibiotic resistance (ARG) accumulated through long-term coevolution and selection [1]. Analysis of DNA from 30,000-year-old permafrost samples revealed functional resistance genes to β-lactams, tetracyclines, and glycopeptides identical to modern clinical variants [2]. Forsberg and colleagues [3] showed that a significant portion of ARGs found in clinical isolates have functional homologs in soil microorganism genomes, with some demonstrating nucleotide sequence identity above 99%—indicating recent horizontal transfer rather than parallel evolution.

In agroecosystems, intensive agricultural activities create selective pressure that can lead to the formation of ARG clusters on mobile genetic elements [4]. These activities include application of organic fertilizers containing antibiotic residues (animal manure and sewage sludge), wastewater irrigation, veterinary antibiotic use, and heavy metal accumulation. The proportion of antibiotic resistance genes can increase by 1–2 orders of magnitude compared to background values in adjacent areas [5]. Under such multifactorial pressure, the horizontal transfer of resistance genes between taxonomically distant groups of microorganisms becomes particularly important, including the possibility of their transmission from soil saprophytes to clinically significant bacteria. Documented cases of such transfer include: NDM metallo-β-lactamase dissemination through soil isolates of *Acinetobacter* and *Enterobacteriaceae* [6]; colistin resistance gene *mcr-1* transition from livestock gut microbiota to clinical *Escherichia coli* and *Klebsiella pneumoniae* [7]; and tetracycline resistance gene migration from soil actinomycetes to pathogens via mobile integrons [3]. These documented transfer routes provide the epidemiological rationale for characterizing both resistome composition and mobilization infrastructure in agricultural soils, which is the central focus of the present study. Transmission from the soil reservoir to humans occurs via several parallel routes: direct contact with soil and plant products, consumption of livestock products, contaminated water sources, and aerosol transfer [8]. Beyond the immediate sites of application, ARGs in agroecosystems are subject to wider environmental dissemination through surface runoff, leaching into groundwater, and aerosol dispersal, enabling accumulation in non-target habitats distant from agricultural fields. ARGs can persist in soils long after the cessation of selective pressure. This persistence is facilitated by physical association with mobile genetic elements carrying toxin–antitoxin maintenance systems, which ensure their retention within microbial communities independently of ongoing antibiotic selection [9]. Thus, the study of soil microbiome resistomes extends beyond ecological microbiology and has direct relevance for infectious disease epidemiology.

West Siberia is of particular interest for agroecosystem resistome research: it is one of Russia’s largest grain-producing regions with developed livestock farming [10], diverse soil types—from leached chernozems to meadow-chernozem and saline soils—and a combination of grain and row crops. These regional characteristics create conditions for anthropogenic pressure on soil microbiomes (organic fertilizers, veterinary antibiotics) and make assessment of ARG transmission risks through ‘soil–food–human’ chains especially relevant [11].

Despite the accumulated body of data on the global distribution of ARGs in soils [12,13], West Siberian agroecosystems remain largely unstudied from the perspective of resistance metagenomics. In a global analysis of 1088 metagenomes, Zheng et al. (2022) [12] identified Siberia as one of the regions with the greatest predictive uncertainty in ARG load mapping—owing to the scarcity of local observations. The only metagenomic study conducted in geographically comparable conditions to the present work— Priobye forest–steppe in Novosibirsk Oblast [14]—covered functional genes of carbon, nitrogen, and phosphorus metabolism but not the resistome. Naumova et al. characterized the microbiome of southern West Siberian chernozems by analyzing 16S rRNA composition depending on soil treatment type—but ARGs were not included in this work either [15]. Thus, for this region, there is no resistome profile comparable to accumulated international data.

This study addresses three objectives. The first objective was to characterize the taxonomic structure of microbial communities and describe the composition, abundance, and functional affiliation of ARGs in soil microbiomes in southern West Siberian agroecosystems. The second objective was to identify genes and genetic structures with the greatest horizontal transfer potential based on analysis of ARG associations with mobile genetic elements. The third objective was to position the obtained data in a global context by comparing with international and regional datasets and to outline priorities for resistome monitoring in West Siberian agroecosystems.

## 2. Results

### 2.1. Taxonomic Structure of Microbial Communities

#### 2.1.1. Family-Level Composition

**Figure 1:**
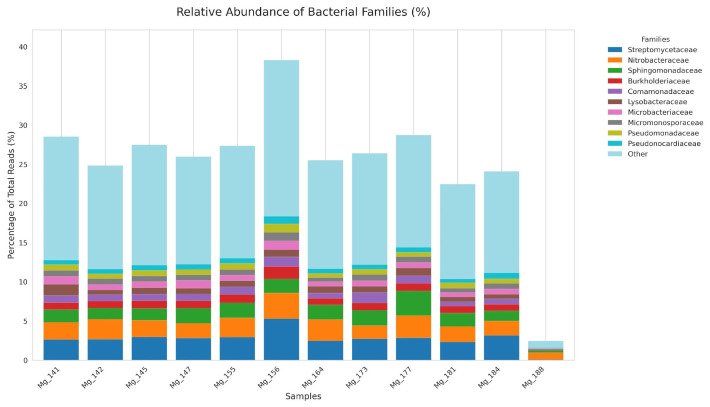
Taxonomic composition of soil microbial communities across 12 metagenomic samples.

Taxonomic classification of microbial sequences using Kraken2 enabled identification of 32.50 to 68.78% of reads (mean 50.64%) in each metagenomic assembly (Figure 1, Table S1). Analysis of distribution at the family level showed that the ten most abundant taxa were detected in all 12 samples, forming a stable core of the agroecosystem’s microbial community. Their total relative abundance varied from 2.05% in Mg_188 to 21.52% in Mg_156, reflecting inter-sample variability primarily in quantitative rather than qualitative terms.

*Streptomycetaceae* showed the highest mean relative abundance across the dataset (mean 2.74%; range 0.07–5.30). It was detected in all 12 samples: the minimum value was recorded in Mg_188 (0.07%), and the maximum in Mg_156 (5.30%), which is 1.8 times higher than the next closest sample Mg_184 (3.15%). *Nitrobacteraceae* ranked second in mean abundance (mean 2.21%; range 0.91–3.28%). As expected for nitrogen-cycling taxa, values were lower in meadow-chernozem samples (Mg_173 1.69%, Mg_181 1.99%, Mg_184 1.86%; mean 1.85%) than in leached chernozems (mean 2.64%, excluding Mg_188). *Sphingomonadaceae* ranked third (mean 1.70%; range 0.25–3.14%); notably elevated relative abundance was recorded in Mg_177 (3.14%, southern chernozem)—1.6-fold higher than the next highest sample.

The fourth most abundant family was *Lysobacteraceae* (mean 0.77%; range 0.09–1.42%), represented in all samples with a maximum value in Mg_141 (1.42%, sandy soils, sunflower). *Burkholderiaceae* demonstrated the most stable distribution across the sample set (0.05–1.60%; mean 0.91%) without pronounced association with soil types or crops, except for the anomalously high value in Mg_156 (1.60%). *Comamonadaceae* were found in all samples except Mg_141 (0%), with a range of 0.10–1.34%; the highest values were characteristic of samples in meadow-chernozem soils (Mg_173—1.34%). The remaining four families—*Pseudomonadaceae*, *Pseudonocardiaceae*, *Phyllobacteriaceae*, and *Microbacteriaceae*—were present in all samples with moderate abundance (0.02–1.13%), showing no pronounced association with specific soil types or crops. Sample Mg_188 was a notable quantitative outlier: the abundance of all ten families was proportionally reduced relative to other samples, yet none of the families were completely absent.

#### 2.1.2. α-Diversity Analysis

**Figure 2:**
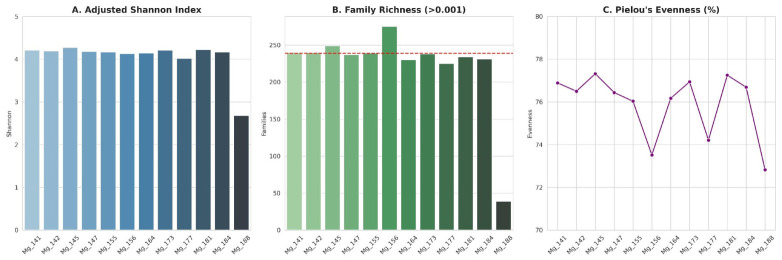
Alpha-diversity of soil microbial communities across 12 metagenomic samples. (**A**) Adjusted Shannon diversity index calculated at the family level (minimum relative abundance threshold 0.001%). (**B**) Number of bacterial families with relative abundance exceeding the threshold. (**C**) Pielou’s evenness index. Horizontal dashed lines indicate the group mean excluding Mg_188.

The adjusted Shannon index (Figure 2, Table S2) varied within a narrow range—from 4.02 to 4.27 in all samples except Mg_188 (2.68). Formal outlier testing confirmed the exceptional status of Mg_188: the Grubbs test applied to Shannon index values yielded G = 3.14, exceeding the critical value of 2.41 (n = 12, α = 0.05). The number of families with occurrence frequency above the threshold value was 225–275 (average 239) with the similar exclusion of Mg_188 (39 families). Pielou’s evenness index varied in the range of 73.52–77.32, indicating uniform distribution of abundance among families in all samples; sample Mg_156 is notable for the combination of the highest number of families (275) and lowest evenness (73.52), which may reflect the presence of several dominant taxa against a background of high richness. The Kruskal–Wallis test revealed no significant difference in Shannon index between meadow-chernozem and other soil types (H = 1.051, *p* = 0.305).

#### 2.1.3. β-Diversity Analysis

**Figure 3:**
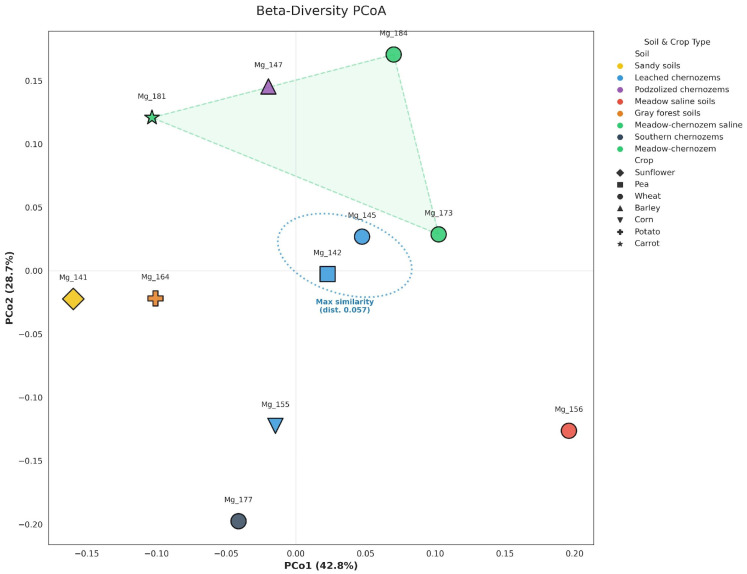
Beta-diversity of soil microbial communities based on Bray–Curtis dissimilarity. Principal Coordinates Analysis (PCoA) of pairwise Bray–Curtis distances computed from family-level relative abundances (Mg_188 excluded). Symbols indicate cultivated crop; colors indicate soil type. The blue circle shows the formation of the cluster with the smallest Bray-Curtis index distance (Mg_142, Mg_145) on one soil type. The green area shows the area that combines the meadow-chernozem soil cluster and the podzolic chernozem soil with an average intra-group Bray-Curtis distance of 0.194 and a reduced content of Nitrobacteraceae.Pairwise Bray–Curtis distances are provided in Table S2.

Mean pairwise Bray–Curtis distance was 0.212 ± 0.069 (range 0.057–0.360). β-diversity was assessed by Principal Coordinates Analysis (PCoA) of the Bray–Curtis pairwise distance matrix for 11 samples (sample Mg_188 was excluded from β-diversity ordination because its anomalously low classification efficiency (32.50% vs. mean 53.18%) would inflate Bray–Curtis distances relative to all other samples) (Figure 3, Table S3). The first two coordinates explained 33.4% (PCo1) and 19.8% (PCo2) of total variation, respectively (cumulative 53.2%). The analysis demonstrated a high degree of similarity among leached chernozem samples, particularly Mg_142 and Mg_145, which showed the minimum pairwise distance in the dataset (0.057, highlighted by a similarity ellipse). A pronounced clustering tendency was observed for meadow soil types (indicated by a polygon), reflecting the compositional coherence of their communities. In contrast, the sandy soil sample (Mg_141) and the meadow solonchak sample (Mg_156) occupied peripheral positions along the first and second axes, reflecting the substantial influence of edaphic factors on the beta-diversity of the soil microbiome. Pairwise Bray–Curtis distances are provided in Table S2.

To test possible structuring factors, mean intra-group and inter-group Bray–Curtis distances were compared under four alternative sample divisions: by crop type (grain vs. non-grain), agrotechnical type (solid sowing vs. row crops), soil type (meadow-chernozem vs. leached chernozems), and geographical location (central and eastern NSO cluster vs. western and southern districts). In none of the variants did inter-group distance exceed intra-group distance: values were 0.210–0.218 and 0.152–0.256 respectively. Slight clustering was observed according to soil type: three samples of meadow-chernozem soils (Mg_173, Mg_181, Mg_184) form a relatively compact cluster with a mean intra-group Bray–Curtis distance of 0.194 and reduced Nitrobacteraceae content (mean 1.85% vs. 2.62% on leached chernozems), while five samples of leached chernozems demonstrate high internal variability with distances from 0.057 to 0.265. An exception is sample Mg_156 (leached chernozem, wheat), which is a consistent outlier in all analyses performed: it had the highest number of families (275), anomalously high Streptomycetaceae content (5.30%), and maximum Bray–Curtis distances to all other samples (0.265–0.336).

Thus, the combination of α-diversity indicators, dominant family composition, and β-diversity provides a consistent picture: the studied agroecosystems are characterized by high and practically uniform taxonomic diversity (Shannon index 4.02–4.27, evenness 73.5–77.3%). The observed inter-sample variability was not explained by the factors tested—crop type, agricultural type, soil type, or geographical location. The absence of significant clustering was confirmed by PERMANOVA: neither crop type (F = 1.791, *p* = 0.145) nor soil type grouping (meadow-chernozem vs. other; F = 1.923, *p* = 0.126) explained a significant proportion of community composition variance (9999 permutations). The available metadata did not include detailed information on fertilization history, pesticide use, or irrigation sources; therefore, the drivers of the residual variability could not be determined within the scope of this study.

### 2.2. General Characterization of the Resistome and Assessment of ARG Load

Genomic annotation yielded ARG quantification data for all 12 metagenomes (Mg No. 141–188), presented in Table 1. The total number of ARGs varied from 249 (Mg_142) to 820 (Mg_145), averaging 520.7 across the sample set. The highest number of unique genes was recorded in sample Mg_188 (49 genes); of these, 20 were represented by a single copy, which is consistent with the high heterogeneity of this sample’s microbiome, confirmed during taxonomic analysis. In other samples, the number of unique genes varied from 18 to 25, and the number of singletons from 2 to 6. For this analysis, unique genes are defined as distinct ARG identifiers (DeepARG gene-level annotation), and singletons as unique genes represented by a single copy in the assembly.

**Figure 4:**
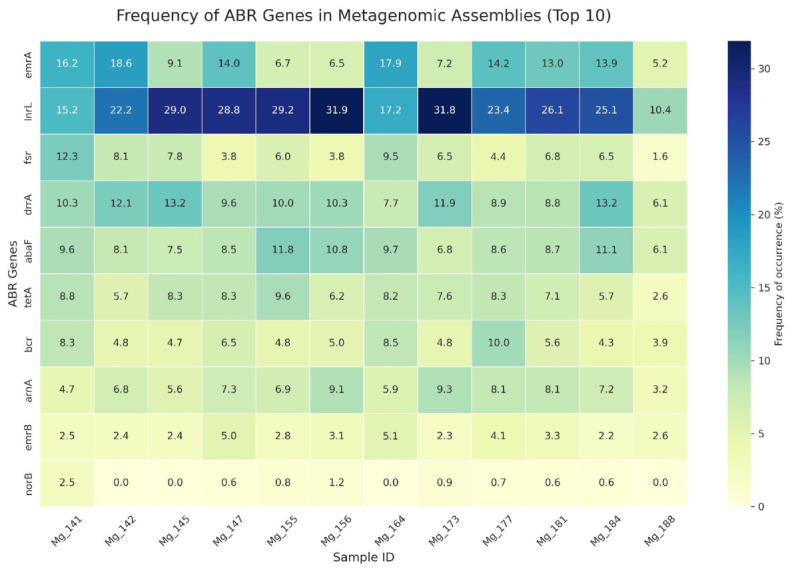
Resistome composition across 12 soil metagenomes. Heatmap of the relative frequency (% of total ARG copies per sample) of the ten most prevalent antibiotic resistance genes identified by DeepARG annotation.

Figure 4 presents the frequency distribution of the 10 most prevalent ARGs (Figure 4, Table S4). The highest average frequencies across the entire sample set were recorded for genes *lnrL* (23.6%) and *emrA* (11.9%). The *lnrL* gene dominates in most samples, reaching maximum values in Mg_156 (31.89%), Mg_173 (31.81%), and Mg_155 (29.18%); and the minimum value in Mg_188 (10.40%). Consistently high values are also demonstrated by genes *drrA* (mean 9.8%), *abaF* (mean 8.9%), and *tetA* (mean 7.2%). The *norB* gene was present in most samples at minimal levels (0.5–2.5%) and was absent in Mg_145, Mg_164, and Mg_188. Functional affiliation analysis (Table 2) shows that efflux pump genes collectively comprise more than half of all detected resistance genes.

#### Normalized Resistome Load and Its Variability

The normalized resistome load (N/*rpoB*) varied from 2.30 (Mg_142) to 5.37 (Mg_147), averaging 3.67 across the sample set (Table 1). Normalization by *rpoB* rather than 16S rRNA was applied to reduce biases from variable gene copy numbers (see Section 4.4.1).

When analyzed by soil type, the trend identified in taxonomic analysis persists: three samples on meadow-chernozem soils (Mg_173—3.88; Mg_181—3.82; Mg_184—3.48) demonstrate similar moderate values for different crops (wheat, carrot, wheat; mean 3.73). Five samples on leached chernozems show a range from 2.30 (Mg_142, pea) to 4.72 (Mg_155, corn), indicating notably greater intra-group variability. Sample Mg_147 (barley, podzolized chernozem) is notable for the highest N/*rpoB* value (5.37) while having the lowest number of rpoB copies (90) among the entire sample set, which may reflect either truly elevated resistome saturation or specific annotation features in this sample. Sample Mg_188 demonstrates the lowest number of *rpoB* copies (126); however, the normalized load indicator (2.45) is comparable to several other samples.

### 2.3. Mobilome and Associations of ARGs with Mobile Genetic Elements

**Figure 5:**
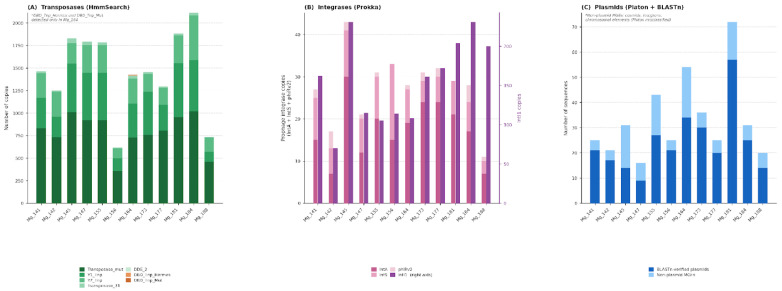
Mobilome composition across 12 West Siberian agroecosystem metagenomes. (**A**) Transposase family distribution detected by profile HMM search (E-value < 10^−9^). (**B**) Prophage integrase families (*IntA*, *IntS*, *phiRv2*; left axis, left bars) and class 1 integron integrase *IntI1* (right axis, right bars) annotated by Prokka. (**C**) Plasmid sequences: BLASTn-verified plasmids and non-plasmid mobile genetic elements reclassified after NCBI homology search. All values are absolute copy numbers per assembly.

All four classes of mobile genetic elements—transposases, prophage integrases, class 1 integron integrases and plasmids—were detected across all 12 samples, indicating consistent mobilome representation throughout the dataset (Figure 5).

#### 2.3.1. Transposases

Predicted transposase sequences were classified into families using profile hidden Markov models (Figure 5, Table S5). Representatives of six families were detected, differing in transposition mechanism and domain organization. The most abundant in all samples were transposases of the Transposase_mut family, whose number varied from 356 (Mg_156) to 1020 (Mg_184); this family includes transposases often associated with IS elements. Also present in significant quantities were transposases of the Y1_Tnp and Y2_Tnp families (from 110 to 599 and from 113 to 495 copies respectively). The highest total number of transposases was recorded in samples Mg_184 (1909 copies) and Mg_164 (1415 copies). Families DBD_Tnp_Hermes and DBD_Tnp_Mut were detected exclusively in sample Mg_164 (gray forest soils, potato), while they were absent in all other samples. The DDE_2 family was also predominantly concentrated in Mg_164 (six copies) but was additionally detected at low abundance in Mg_145 (two copies) and Mg_177 (one copy).

#### 2.3.2. Prophage Integrases

Integrases were detected by homology with three types of proteins: *IntA* (lambda-type phage integrase), *IntS* (P4-type phage integrase), and *phiRv2*. In all analyzed metagenomic assemblies, representatives of all three families were detected (Figure 5, Table S6). The most numerous were integrases of the *IntA* family (from 6 to 30 copies; maximum in Mg_145). *IntA* predominated over *IntS* and *phiRv2*, except for Mg_156, where *IntS* (18 copies) slightly exceeded *IntA* (15 copies). The minimum total content of prophage integrases was recorded in Mg_188 (seven *IntA*, three *IntS*, one *phiRv2*).

#### 2.3.3. Class 1 Integron Integrases (*intI1*)

Using hidden Markov models (threshold E-value < 10^−10^, bit score > 100), class 1 integron integrase (*intI1*) sequences were identified in all 12 samples (Figure 5, Table S6). The normalized load indicator (*intI1*/*rpoB*) varied from 0.64 (Mg_155) to 1.59 (Mg_188), averaging 1.07 across the sample set. The highest values were recorded in samples Mg_188 (1.59), Mg_147 (1.28), and Mg_184 (1.24); the lowest in Mg_155 (0.64) and Mg_142 (0.65). The range of inter-sample variability is relatively small: the coefficient of variation was 24%, while for the total resistome load indicator N/*rpoB* the analogous indicator reaches 31%, indicating more uniform distribution of *intI1* across the sample set compared to the total resistome.

#### 2.3.4. Plasmids

At the primary analysis stage using Platon, plasmid sequences were found in all samples (Figure 5, Table S7). The number of preliminarily detected plasmids varied from 16 (Mg_147) to 72 (Mg_181). After verification using BLASTn, confirmed plasmid sequences numbered from 9 (Mg_147) to 57 (Mg_181); the proportion of confirmed plasmids varied from 67% (Mg_147) to 94% (Mg_156). Samples with the highest number of verified plasmids—Mg_181 (57), Mg_164 (34), and Mg_173 (30)—differ in both crop and soil type, which does not allow us to link high plasmid content to any single factor. In all samples, sequences (from 4 to 20 per sample) were detected that were erroneously classified by Platon as plasmid but were assigned to other types of mobile elements (cosmids, integrons, chromosomal elements) based on BLASTn results.

#### 2.3.5. ARG-MGE Associations

**Figure 6:**
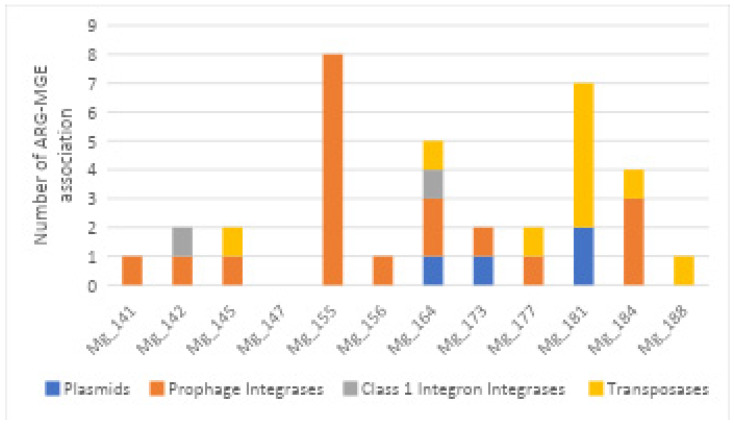
ARG–mobile genetic element associations across 12 soil metagenomes.

Antibiotic resistance genes are associated with mobile elements in 11 of 12 studied metagenomes; the only sample without associations was Mg_147 (barley, podzolized chernozem) (Figure 6). The total number of verified associations varied from one to eight; the highest number of events was recorded in Mg_155 (eight; leached chernozem, corn) and Mg_181 (seven; meadow-chernozem, carrot). Samples with zero or single associations include both grain crops (Mg_147, Mg_156, Mg_188, Mg_142) and non-grain crops (Mg_141).

Analysis of MGE types shows pronounced heterogeneity of dominant mobilization vectors between samples. Integrases absolutely dominate in Mg_155 (eight of eight associations) and predominate in Mg_164 (three of five) and Mg_184 (three of four). Transposases are the main vector in Mg_181 (five of seven) and the only one in Mg_188 (one of one). Plasmids participate in associations in three samples (Mg_164, Mg_173, Mg_181), but in none are they the dominant vector.

A key observation is the diversity of mobilization mechanisms for the same genes: the *emrA* gene was found in association with integrases in Mg_155; with plasmids in Mg_164, Mg_173, and Mg_181; and with transposases in Mg_145, Mg_181, and Mg_184. The *bcr* gene was found in association with integrases in Mg_155 and Mg_173, and with transposases and plasmid in Mg_181. A similar pattern was described by Jechalke et al. for sulfonamide-resistant genes in soils fertilized with manure [16]. Detailed analysis of associations at the level of individual loci is presented in Table 3 and Table S8.

## 3. Discussion

### 3.1. Taxonomic Structure of Agricultural Microbial Communities: Composition Stability and Regional Context

Taxonomic profiling of 12 samples revealed a stable microbial core consistently present across all samples: ten families were detected in every sample, with their total proportion in classified sequences varying primarily quantitatively rather than qualitatively (2.05–21.52%). The community was dominated by *Streptomycetaceae* (mean 2.74%), *Nitrobacteraceae* (2.21%), and *Sphingomonadaceae* (1.70%), followed by *Lysobacteraceae*, *Burkholderiaceae*, *Comamonadaceae*, *Pseudomonadaceae*, *Pseudonocardiaceae*, *Phyllobacteriaceae*, and *Microbacteriaceae*. The Shannon index varied within a narrow range of 4.02–4.27, and the Pielou evenness index within 73.5–77.3%, indicating high and practically uniform taxonomic diversity.

β-diversity analysis based on the Bray–Curtis pairwise distance matrix (mean 0.212; range 0.057–0.360) revealed no significant clustering by any of the tested factors—type of cultivated crop (cereal vs. non-cereal), agricultural type (continuous seeding vs. row crops), soil type, or geographical location. In none of these comparisons did the inter-group Bray–Curtis distance exceed the intra-group distance (values 0.210–0.218 vs. 0.152–0.256, respectively). Thus, none of the tested categorical factors—crop type, agricultural practice type, soil type, or geographic location—were significant structuring variables for microbial community composition. The residual inter-sample variability likely reflects site-specific edaphic and land-use history factors that were beyond the scope of the metadata collected in this study.

Against the background of general sample uniformity, two samples stand out. Sample Mg_188 was a quantitative outlier across multiple metrics: classified sequence proportion 32.50% vs. mean 53.18% for remaining samples; Shannon index 2.68, more than three standard deviations below the dataset mean; and 39 families above threshold vs. 225–275 in other samples. Notably, none of the ten leading families in Mg_188 is completely absent: the qualitative composition of the community core is preserved; only its quantitative representation changes. Standard sequencing QC metrics for Mg_188 (filtering efficiency 92.67%, N50 708 bp, contig count 550,620) were within acceptable ranges, providing no technical basis for excluding this sample from the dataset.

Sample Mg_156 (meadow saline and alkaline soils, wheat) showed the highest number of families in the sample set (275), anomalously high *Streptomycetaceae* content (5.30%)—1.8 times higher than the next highest sample—and the greatest pairwise Bray–Curtis distances to all other samples (0.265–0.336). This pattern is consistent with the specific conditions of saline soils—elevated pH, special ionic composition, and water regime—favoring actinomycete taxa with developed mechanisms of osmotic and chemical adaptation [17].

Comparison with the nearest regional data shows qualitative similarity in taxonomic structure. [15], who studied bacterial biomes of Novosibirsk Oblast chernozems using V3/V4 16S rRNA amplicon sequencing, recorded a dominance of *Actinomycetota* (on average 51% of sequences), *Acidobacteriota*, and *Pseudomonadota*, collectively comprising about 80% of the community. In this work, leading families at the metagenomic profiling level also belong to these same three phyla. The difference in *Actinomycetota* abundance between the two studies is primarily methodological: amplicon sequencing introduces systematic biases in phylum-level abundance estimates compared to shotgun metagenomics [18,19], with the magnitude of this bias depending on the primer pair used. The fundamental similarity consists in the fact that chernozem agroecosystems of southern West Siberia are characterized by stable dominance of the same major taxa regardless of agricultural practices and cultivated crops [15].

The stability of the taxonomic core of the studied agroecosystems—primarily the stable dominance of *Streptomycetaceae* in all 12 samples—means that part of the observed diversity of ARGs is a predictable consequence of this composition, rather than exclusively a result of anthropogenic enrichment.

The present study has several limitations that should be acknowledged. First, the dataset comprises 12 samples collected in a single campaign (September 2022), which constrains both statistical power and temporal generalizability. Seasonal dynamics of soil resistomes—including spring peaks associated with snowmelt and fertilizer application, and autumn shifts following harvest—remain uncharacterized for this region. Second, the spatial coverage, while spanning several districts of Novosibirsk Oblast, does not fully represent the diversity of West Siberian agroecosystems, which extend across multiple oblasts with contrasting climatic and pedological conditions. Future studies should prioritize: (i) increased sample sizes across broader geographic and soil-type gradients; (ii) seasonal sampling designs to capture temporal variability in ARG abundance and MGE activity; and (iii) paired sampling of agricultural and adjacent undisturbed soils to establish site-specific background values.

### 3.2. Regional Resistome Dominated by Widely Distributed Efflux Genes

Analysis of the resistome structure revealed a predominance of genes related to efflux mechanisms (*emrA*, *drrA*, *tetA*, *abaF*, *bcr*, *fsr*), as well as target modification (*lnrL*) and lipid A modification genes (*arnA*); collectively, efflux pump genes constitute more than half of all detected resistance genes. However, by frequency of individual gene occurrence, the gene with the highest mean frequency was not an efflux representative but the target modification gene *lnrL* (mean 23.6%). This observation is consistent with data by McCann et al. for isolated Antarctic soils [20] and a global analysis of 1088 metagenomes by [12], both reporting a predominance of efflux-related ARGs across diverse soil environments. Armalytė et al. confirmed the dominance of RND and ABC transporters in soil isolates from both organic and conventional farming systems [21].

The similarity of predominant resistance mechanisms in ecosystems with markedly contrasting conditions—from temperate agroecosystems to isolated Antarctic environments—indicates the fundamental role of efflux systems in microbial community adaptation to stress factors of both anthropogenic and natural origin [22,23]. Ref. [5], analyzing resistomes of soil communities from nine different habitats, established that functional categories of ARGs—including efflux transporters—are taxonomically determined and reproducible within a phylum regardless of anthropogenic pressure, explaining their global constancy in soil metagenomes [5]. Nevertheless, the dominance of efflux genes per se is not an indicator of anthropogenic enrichment: efflux pumps perform a wide spectrum of physiological functions, including protection from plant toxins and heavy metals, and are present in natural soils independent of agricultural use [24]. The resistome of the studied agroecosystems of southern West Siberia is consistent with this global pattern.

The normalized resistome load N/*rpoB* ranged from 2.30 to 5.37 (mean 3.67). For context, published N/*rpoB* reference ranges include: 2.1–4.8 (median 3.2) for soils under mineral fertilization only [13]; up to 9.3 for soils receiving regular untreated manure [13]; ~3.5 as the global median for temperate agricultural soils [12]; and 0.8–1.9 for near-pristine environments [20,25]). The mean N/*rpoB* of 3.67 obtained in this study falls within the range characteristic of agroecosystems receiving mineral fertilizers without regular manure application, and is approximately 2.5-fold below values reported for intensively manured soils. This indicates a moderate level of anthropogenic resistome enrichment in the studied agroecosystems, although direct quantitative comparison is limited by differences in the normalization methodology between studies.

The *lnrL* gene ranked first by mean frequency of individual gene occurrence across all samples (23.6%), significantly exceeding the next most prevalent gene, *emrA* (11.9%). The *lnrL* gene encodes an ATP-binding protein of ABC transporter providing resistance to lincosamides and oxazolidinones [26]. Linezolid—a representative of the latter class—is a reserve drug for treating infections caused by vancomycin-resistant enterococci and MRSA. From a risk assessment perspective, two scenarios for *lnrL* presence in soil must be distinguished. Genes of the *lnr* family are widely distributed among soil actinomycetes, primarily in *Streptomyces*, where they are part of biosynthetic clusters and perform self-protection functions against their own antibiotics [27]. High content of Streptomycetaceae in all studied samples is consistent with this explanation and suggests a natural rather than anthropogenic source for most *lnrL* copies.

The second most frequent gene in the sample set, *emrA* (mean 11.9%; range 5.20–18.55%), as well as functionally coupled *emrB* (mean 3.08%), encode components of the three-component efflux pump EmrAB-TolC belonging to the MFS superfamily. Membrane adaptor *EmrA* and transporter *EmrB* form a functional heterodimer [28] providing efflux of nalidixic acid, novobiocin, and several detergents. Unlike clinical resistance genes with pronounced anthropogenic signal, *emrAB* genes are widely distributed among Gram-negative soil bacteria [29] as part of the basic efflux repertoire: they occur in *Pseudomonas*, *Bradyrhizobium*, *Sphingomonas*, and several Bacteroidota, i.e., in taxa dominating the studied samples. This explains their high total frequency: it primarily reflects the taxonomic composition of the community rather than indicating anthropogenic enrichment. Nevertheless, the wide distribution of *emrA* in several taxonomic groups simultaneously creates prerequisites for frequent horizontal events: the greater the number of taxa carrying original gene copies, the higher the probability of finding it on mobile platforms in new hosts. This hypothesis is directly supported by the MGE association data discussed below.

Detection of the *arnA* gene in 11 of 12 samples with frequencies up to 9.3% also requires distinguishing between two sources. The most likely anthropogenic pathway is application of organic fertilizers: colistin was widely used in veterinary medicine, and according to Touati et al. [30], are livestock feces. *ArnA* (ArnBCADTEF system) is a chromosomal gene widely distributed among Gram-negative soil bacteria (*Pseudomonas*, *Burkholderia*, *Rhizobium*) as part of adaptation to cationic antimicrobial peptides [31]. Its high occurrence in all samples regardless of the crop type and soil is consistent precisely with the natural pool. Zheng et al. detected polymyxin resistance genes even in soils with minimal anthropogenic impact, additionally confirming the existence of a natural reservoir of these determinants [12].

### 3.3. ARG Subset Physically Associated with Different Classes of Mobile Genetic Elements

A key finding of the study was the identification of ARG associations with plasmids, integrases, and transposases in 11 of 12 samples. The obtained data are consistent with conclusions by Deng et al. [13], who showed, based on analysis of 180 soil metagenomes from different land use types, that plasmids make a major contribution to the horizontal transfer of ARGs in agricultural soils, and the relative abundance of MGEs reaches maximum values in agroecosystems. Ref. [32], based on a global sample, showed that the same ARGs can be transferred by fundamentally different vectors depending on the ecological context.

The highest number of ARG-MGE associations was recorded in samples Mg_155 (eight events) and Mg_181 (seven events). In Mg_155, associations with integrases absolutely dominate (eight of eight events), indicating the leading role of prophage transduction in this community. In Mg_181, conversely, transposases are the leading vector (five of seven events), while plasmids make an additional contribution (two events). Transposases also predominate in Mg_188 (one of one). In other samples with multiple associations—Mg_164 and Mg_184—integrases dominate. Thus, the type of dominant mobilization mechanism was determined neither by the crop type nor by the soil type, and apparently reflects the local history of a specific field and its microbial community composition.

Sample Mg_147 (barley, podzolized chernozem) deserves special attention, as it demonstrates the highest normalized resistome load among the samples in the set (N/*rpoB* = 5.37) and a complete absence of ARG-MGE associations. This apparent paradox may be explained by several alternative interpretations. First, high resistome load may be formed predominantly by chromosomal determinants evolutionarily not integrated into mobile platforms. Second, Mg_147 is characterized by the lowest number of *rpoB* copies in the sample set (90), indicating relatively low assembly coverage depth: short contigs may not capture physical co-localization of ARG and MGE. Regardless of the cause, Mg_147 clearly illustrates that high total ARG abundance per se is not a sufficient indicator of horizontal spread risk.

**Figure 7:**
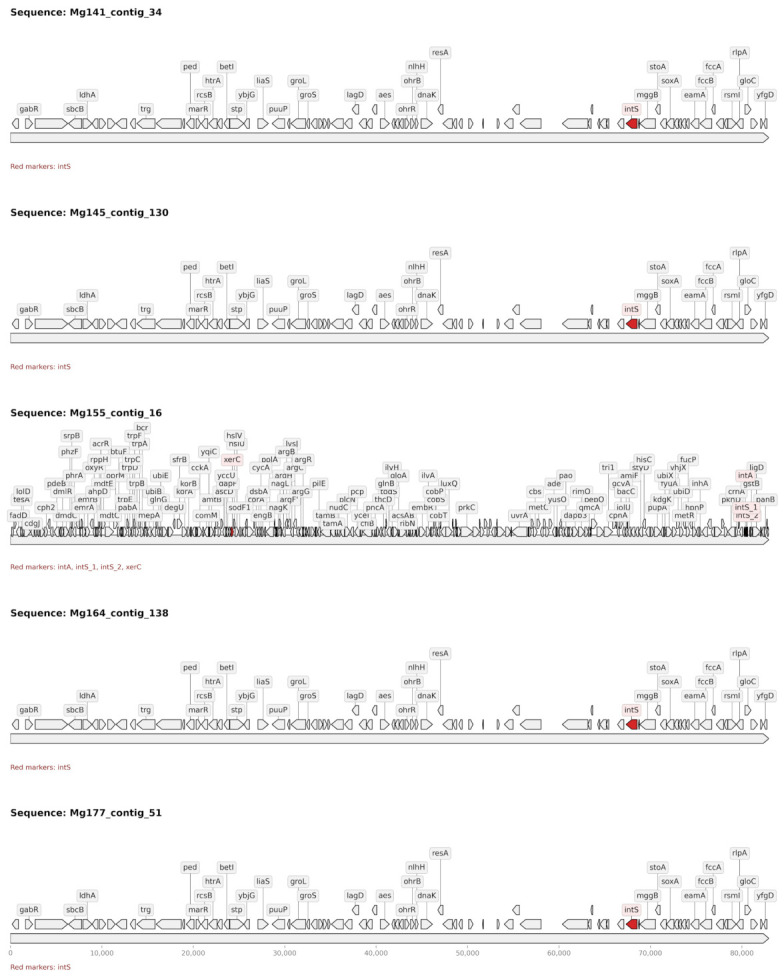
Syntenic organization of *MarR*-containing integrative elements across West Siberian soil samples. The 83 kb elements display two distinct architectural patterns: four samples (Mg_141, Mg_145, Mg_164, Mg_177) show conserved modular organization with (i) 5′-terminal transcriptional regulation region (*gabR*, *sbcB*, *trg*, *MarR*), (ii) central metabolic core (*ped*, *bet1*, *htrA*, *rcsB*, *stp*, *ybjG*, *groL*, *groS*), and (iii) 3′-terminal mobile element machinery (IntS integrase, *stoA*, *soxA*, *fccB*, *glpC*, *mgpB*, *eamA*, *rsmI*, *yfgD*), while sample Mg_155 exhibits a complex mosaic structure with multiple integrase families (*IntA*, *IntS_1*, *IntS_2*, *xerC*) and expanded gene repertoire. Red markers indicate integrase genes. Scale bar represents nucleotide positions (bp). All elements maintain identical length (82,970 bp) despite internal structural variation.

A highly conserved 83 kb integrative mobile genetic element carrying *MarR* transcriptional regulators was identified in 5 of 12 analyzed soil samples (Mg_141, Mg_145, Mg_155, Mg_164, and Mg_177). While maintaining an identical length (82,970 bp) across all detection sites, the elements displayed two distinct organizational patterns (Figure 7): a simpler structure observed in four samples (Mg_141, Mg_145, Mg_164, Mg_177) featuring basic *MarR* regulators and single class S integrase genes (*IntS*), and a more complex mosaic architecture in sample Mg_155 containing multiple integrase families (*IntA*, *IntS*, *XerC*) and an expanded gene repertoire. Comparative genomic analysis confirmed association with soil-dwelling *Ralstonia* species (*R. mannitolilytica*, *R. pickettii*). The presence of site-specific integrases without detectable autonomous conjugation machinery suggests a classification as Integrative and Mobilizable Elements (IMEs) capable of stress-inducible horizontal transfer [33]. MarR systems typically regulate efflux pumps that confer resistance to aromatic compounds, organic solvents, and oxidative stress agents [34,35]. The widespread distribution of these elements may reflect selective pressure from chemical compounds consistent with the documented substrate specificity of MarR regulators, which respond to aromatic compounds, organic solvents, and oxidative stress agents. However, no measurements of such compounds were performed in this study, and any connection to specific agricultural inputs in the studied agroecosystems remains hypothetical. This represents the first documented widespread distribution of *MarR*-containing integrative elements in agricultural soil bacterial communities, with evidence of both conserved and mosaic structural variants co-occurring in the same geographic region.

Notably, the same ARGs were found to be associated with distinct classes of mobile elements across different samples (Table 3). This MGE polymorphism—a single gene mobilized via integrases, plasmids, and transposases in different samples—parallels the pattern described by Jechalke et al. [16] for sulfonamide resistance genes in manure-fertilized soils, and suggests that multiple independent transfer routes operate in parallel within this agroecosystem region. *emrA* proved to be the most “mobilization-active” gene of the entire sample set: it was found in association with MGE in seven of 12 samples, utilizing all three detected classes of mobile elements—integrases (Mg_155), plasmids (Mg_164, Mg_173, Mg_181), and transposases (Mg_145, Mg_181, Mg_184)—which was not observed for any other gene in the sample set. Such “MGE polymorphism” is consistent with EmrA biology: the protein functions together with different outer membrane channels and inner transporters [36]; therefore, its gene may retain functionality during horizontal transfer to taxonomically distant hosts without the mandatory co-transport of partner subunits. Remarkably, in sample Mg_181, *emrA* is associated with both a plasmid and a transposase, indicating that at least two independent mobilization mechanisms operate in parallel within a single community.

**Figure 8:**
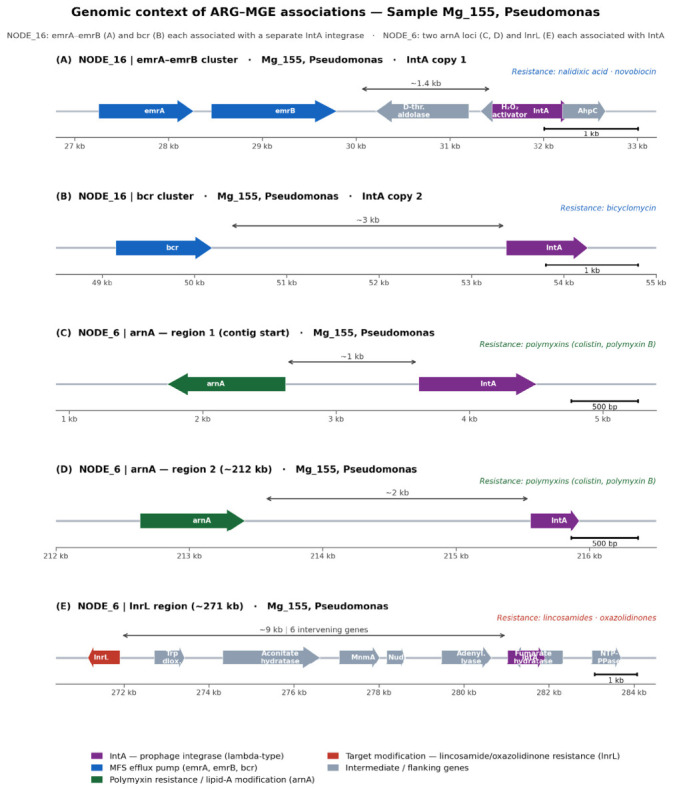
Genomic context of ARG–MGE associations in sample Mg_155 (*Pseudomonas*). Linear contig maps drawn to scale for NODE_16 (panels (**A**,**B**)) and NODE_6 (panels (**C**–**E**)).The direction of the arrows in this figure indicates the direction of gene transcription.The alignment of the orientation and position of the predicted gene with the intA integrase is shown by superimposed arrows.

We additionally identified cases of co-localization of several ARGs on a single contig in association with prophage integrase, identified in sample Mg_155 (Figure 8). On contig NODE_16, genes *emrA* (27,256–28,452 bp) and *emrB* (28,458–30,035 bp) are co-localized within ~2 kb of IntA copy 1 (~32 kb), while *bcr* (49,150–50,376 bp) is independently associated with a second IntA copy (~53 kb) located ~19 kb downstream—indicating two distinct horizontal transfer events on the same contig. On contig NODE_6 of the same sample, two consecutive *arnA* loci (regions 1 and 2, separated by ~206 kb of contig sequence) and the *lnrL* gene (~271 kb) each occur in independent association with IntA copies, with intergenic distances of ~1 kb, ~2 kb, and ~9 kb respectively.

Detection of three families of prophage integrases (IntA, IntS, phiRv2) indicates active lysogenization processes in the studied microbial communities. Santos et al. [25] emphasized the role of phage-associated mobile elements in ARG gene dissemination even under conditions of minimal anthropogenic impact; our data are consistent with this conclusion and demonstrate that the contribution of conjugative transfer (plasmids) and transduction (phages) varies depending on the specific sample. The predominance of prophage integrases as the leading ARG mobilization vector—observed in 6 of 11 samples with detected ARG–MGE associations—contrasts with the plasmid-centric view that dominates the current literature on agricultural soil resistomes [13,32]. While plasmid-mediated conjugation is typically considered the primary driver of horizontal ARG transfer in agroecosystems, our data suggest that phage-mediated transduction may be equally or more important in systems with lower anthropogenic pressure, where plasmid-carrying taxa are less enriched.

### 3.4. Class 1 Integron Integrases as Markers of Anthropogenic Pressure

The obtained data on intI1 allow direct characterization of the mobilization potential of the studied agroecosystems in terms of anthropogenic resistome enrichment. IntI1 was detected in all 12 samples; the normalized *intI1*/*rpoB* ratio ranged from 0.64 to 1.59 (mean 1.07) and showed no significant correlation with either total resistome load (N/*rpoB*) or the number of ARG-MGE associations. This observation is consistent with the position of Gilling et al. [37], emphasizing that intI1 reflects the general level of anthropogenic selective pressure on the microbial community rather than the current frequency of the horizontal transfer of specific ARGs.

The key observation is that, in 9 out of 12 samples, the *intI1*/*rpoB* value is greater than one (average value 1.07, range 0.64–1.59). Since *rpoB* is a single-copy marker gene [38], this means that in metagenomic assemblies for each “bacterial unit” identified through *rpoB*, there is on average more than one copy of the class 1 integron integrase—which is possible with high proportional representation of *intI1*-carrying taxa or with the presence of multiple copies of the *intI1* per genome. Gillings et al. showed that *intI1*/*rpoB* values above unity are characteristic of soils experiencing chronic load of organic fertilizers or wastewater [37]. Similar values were recorded by Jechalke et al. in fields regularly receiving manure from animals prescribed antibiotics [16]. Together with moderate N/*rpoB* values and the presence of ARG-MGE associations in 11 of 12 samples, *intI1* abundance > 1 indicates established stable mobilization infrastructure in regional agroecosystems, exceeding the expected natural background.

Sample Mg_188 warrants particular attention, as it combined the highest *intI1*/*rpoB* value in the dataset (1.59) with minimal taxonomic diversity, low normalized resistome load, and a complete absence of ARG–MGE associations. This combination is consistent with the interpretation that high intI1 abundance reflects a recent anthropogenic impact—the probable application of organic fertilizers—that introduced integron-carrying bacteria into soil without their stable establishment in the local community. However, direct confirmation through field application records or antibiotic residue measurements was not available in the present study, and this interpretation remains inferential. Transient *intI1* elevation after manure application, not accompanied by a proportional increase in ARG-MGE associations, was described by Jechalke et al. [16] as a “tidal” effect of the exogenous resistome dispersing over several months in the absence of repeated input. Mg_188 may represent exactly such a transient stage. A contrasting pattern was observed in sample Mg_155: the lowest *intI1*/*rpoB* (0.64) with the maximum number of ARG associations with integrases (eight of eight). This indicates that in this sample, horizontal gene transfer occurs predominantly through prophage transduction rather than via conjugative platforms of class 1 integrons—two fundamentally different mobilization vectors that can substitute for each other depending on microbial community composition.

At a homology threshold of ≥80%, a dihydrofolate reductase gene—*dfrA1* (98.7% identity with a secondary match to *dfrA16* at 80.1% reflecting database cross-reactivity between closely related variants)—was co-localized with *intI1* on contig NODE_45769 in sample Mg_164 (gray forest soils, potato), at a distance of approximately 4.9 kb. This gene encodes a trimethoprim-resistant DHFR variant and represents one of the most prevalent cassettes in class 1 integrons of clinical isolates worldwide [39,40]. The 98.7% identity to its clinical reference indicates that the soil reservoir in Mg_164 already harbors a functionally equivalent—rather than merely ancestral—form of this mobilized cassette. A tetracycline resistance gene *tet(64)* (84.0% identity, MFS efflux) was additionally detected in association with intI1 in sample Mg_142 (NODE_2278419, ~4.9 kb distance). This finding is particularly relevant given that Mg_142 carries the lowest overall resistome load in the dataset (N/*rpoB* = 2.30; *intI1*/*rpoB* = 0.65), demonstrating that minimal bulk ARG abundance does not preclude the presence of mobilized, clinically relevant determinants—a distinction with direct implications for surveillance design.

### 3.5. Natural Origin Determinants on Mobile Platforms: Individual Gene Analysis

In addition to mobilization markers, specific ARGs whose epidemiological significance depends not on total abundance but on evidence of transfer to new hosts warrant separate consideration. Results of genomic annotation revealed in the studied agroecosystems a set of ARGs with a documented natural actinomycete or soil origin: *bcr*, *drrA*, *fsr*, *lnrL*, *ble*, *marR* [41]. However, for each of these determinants, the epidemiological significance is determined not by presence in the soil metagenome per se, but by the fact of association with MGE in taxonomically distant hosts. As long as a gene remains confined to its original actinomycete host and performs a self-protection function, its detection in the soil resistome constitutes a background ecological signal [42]. When the same gene is identified in Gram-negative soil bacteria in association with an integrase, plasmid, or transposase, this documents confirmed or ongoing transfer beyond the natural pool. This distinction frames the following analysis.

Taxonomic attribution of contigs carrying *lnrL* in association with MGE revealed a fundamentally important pattern in two samples. In Mg_155, the gene was found in Pseudomonas in association with prophage integrase IntA; and in Mg_188 in a taxonomically unattributed contig in association with Transposase_mut family transposase. Pseudomonas is a widely distributed soil bacterium including clinically and phytopathogenically significant species. The presence of *lnrL* in a *Pseudomonadota* contig associated with prophage integrase suggests that horizontal transfer of this gene beyond its natural actinomycete host has occurred in at least one of the studied agroecosystems. This increases potential risk assessment compared to a scenario where *lnrL* remains confined to *Streptomyces* genomes.

The *bcr* gene was detected in all 12 samples (3.9–10.03%); the protein Bcr which it encodes belongs to the MFS superfamily and functions as an efflux pump providing bicyclomycin efflux from the cell [43]. The wide distribution of *bcr* is explained by two sources. On one hand, the gene is part of a chromosomal locus present in the vast majority of Gram-negative genomes as a basic determinant of natural resistance [43]. On the other hand, bicyclomycin biosynthesis clusters have been found in several *Pseudomonadota*, including *Pseudomonas aeruginosa*, with Vior et al. [44] directly stating that *bcm*/*bcr* clusters in *Pseudomonadota* are “almost always associated with mobile genetic elements.” This literature observation receives direct confirmation in this work: *bcr* is associated with integrases in Mg_155 and Mg_173 and with transposase together with plasmid simultaneously in Mg_181—characterizing it as one of the most mobilization-active resistome components regardless of the soil type or crop. The clinical significance of these data is determined by the current renaissance of bicyclomycin as a therapeutic candidate: in combination with doxycycline it showed synergistic lethality in vitro and in mouse infection models against carbapenem-resistant *Enterobacteriaceae* and ESBL-producing *E. coli* and *Klebsiella pneumoniae* [45]. The widespread distribution of a resistance gene against a potential reserve antibiotic in the soil mobilome of agroecosystems should be considered when evaluating the clinical prospects of bicyclomycin.

The *drrA* gene, encoding the ATP-binding component of ABC transporter with anthracycline efflux (doxorubicin, daunorubicin), ranked third by mean frequency of individual gene occurrence in the sample (9.8%). Anthracyclines are key antitumor drugs; high frequency of *drrA* in soil metagenomes primarily reflects the presence of streptomycete anthracycline producers, in whose genomes this gene performs a self-protection function [46]. In sample Mg_184 (meadow-chernozem, wheat), *drrA* was found in association with prophage integrase *IntA* on a contig attributed to *Bradyrhizobium*, indicating its potential for horizontal transfer beyond the actinomycete pool. As a nitrogen-fixing rhizobium widely distributed in agroecosystem rhizospheres [47], *Bradyrhizobium* as a recipient of *drrA* represents a potential link between the streptomycete reservoir and the crop rhizosphere. The clinical significance of anthracyclines as cancer chemotherapy agents makes monitoring of mobile *drrA* variants advisable in epidemiological surveillance programs.

Fosfomycin (phosphomycin) is a natural antibiotic of a streptomycete origin. The Fsr protein of *Sinorhizobium meliloti* is an MFS transporter providing fosfomycin efflux; unlike mobile *fosA* determinants in Enterobacteriaceae, actively transferred by plasmids and integrons [48], *fsr* is chromosomally fixed in rhizobia and performs a multifunctional role: besides fosfomycin resistance, it participates in biofilm regulation, motility, and symbiotic efficiency with legumes [49]. This explains the high background abundance of *fsr* in all samples—a reflection of rhizobial taxa rather than antibiotic pressure—and the absence of its associations with MGE in this sample. Sample Mg_141 (sandy soils, sunflower) with an anomalously high *fsr* proportion (12.29%) is characterized by unique soil substrate with probable enrichment of rhizobial taxa. In contrast, data from *Ralstonia* reveal a different pattern: in Mg_177, the *abaF* gene (fosfomycin resistance, originally characterized in *Acinetobacter baumannii*) was detected in *Ralstonia* in association with Transposase_mut, and in Mg_181, a fosfomycin resistance protein gene in *Ralstonia* was associated with Transposase_31. Thus, while *fsr* in rhizobia retains chromosomal status, homologous determinants in *Ralstonia solanacearum*—a phytopathogen with a broad host range among cultivated plants—are already located on transposase platforms, documenting successful horizontal transfer within the agroecosystem compartment.

The *ble* gene (bleomycin resistance) has a natural origin in actinomycetes; in sample Mg_164 it was found in association with Y1_Tnp family transposase on a contig attributed to *Mucilaginibacter*, indicating its mobilization potential in this soil community and demonstrating consistency with previous reports of *ble* association with MGEs in clinical isolates [50].

Comparison of natural determinant distribution on mobile platforms in this sample with global data reveals both similarities and notable differences. Santos et al., studying Antarctic soils with minimal anthropogenic impact, recorded a similar pattern: natural determinants of an actinomycete origin (including *lnr* and *bcr* family genes) predominated in the resistome, but their associations with MGE were rare [25]. Conversely, in Chinese agricultural soils, the proportion of mobilized natural determinants was substantially higher, with plasmids serving as the dominant vector [13]. Based on the available comparative data, the studied West Siberian agroecosystems appear to occupy an intermediate position: natural determinants form the resistome basis, but their associations with MGEs—primarily with prophage integrases in *Pseudomonas* and *Bradyrhizobium*—are consistent with active mobilization processes rather than a purely background state. We acknowledge that this comparison is based on a limited number of studies with heterogeneous methodologies, and broader generalizations should be made with caution.

## 4. Materials and Methods

### 4.1. Sample Collection and Study Objects

**Figure 9:**
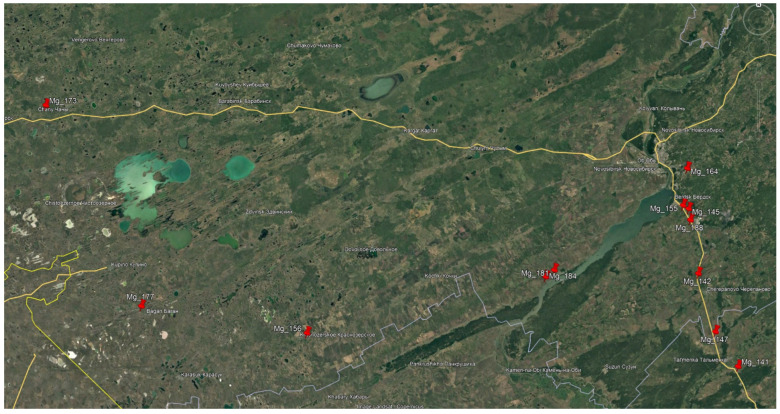
Coordinates of sampling points.

Soil samples from agroecosystems were collected in southern West Siberia in September 2022 according to Russian national standard GOST 17.4.4.02-84 [51], using the composite ‘envelope’ sampling pattern (five sub-samples per plot arranged in a cross pattern, pooled into a single composite sample). The study objects were soil samples from fields occupied by the following agricultural crops: wheat, barley, peas, potatoes, carrots, sunflower, and corn. The soil type during collection was determined as ‘chernozem soils.’ Soil types were subsequently verified using the ‘Soil-Geographic Database of Russia’ information system (https://soil-db.ru/map, accessed on 10 April 2026). Coordinates of sampling points are presented in Figure 9, and sample characteristics are shown in Table 4.

### 4.2. DNA Extraction

Total genomic DNA was extracted from 250 mg of soil samples using the MagMAX Microbiome Ultra Nucleic Acid Isolation Kit (Thermo Fisher Scientific, Austin, TX, USA) following the manufacturer’s protocol. Soil samples were lysed using specialized bead tubes with mechanical disruption for 10 min, followed by thermal and enzymatic lysis. DNA purification was performed using magnetic bead-based technology with wash steps to remove PCR inhibitors and humic substances. DNA was eluted in 50 μL of nuclease-free water.

DNA concentration and purity were determined using a NanoDrop 2000 spectrophotometer (Thermo Scientific, USA). Quality was assessed by A_260_/A_280_ and A_260_/A_230_ ratios, and DNA integrity was verified by agarose gel electrophoresis. Extracted DNA was stored at –20 °C prior to downstream applications.

### 4.3. Sequencing

Metagenomic libraries were prepared using the NEBNext Ultra II DNA Library Prep Kit for Illumina (NEB) with some modifications to the manufacturer’s protocol. A total of 15 ng of metagenomic DNA was fragmented to 400–500 bp on a Covaris S220 (Woburn, MA, USA) instrument using microTUBE-50 AFA Fiber Screw-Cap tubes (Covaris) in 50 or 100 μL of sterile water. Fragmented DNA was cleaned and concentrated using AMPure XP magnetic beads (Beckman Coulter (Brea, CA, USA)), with beads mixed with DNA in a 1.6:1 ratio. The cleaned and fragmented DNA was used in end repair, 3′-end adenylation, and NEBNext Adaptor for Illumina (NEB) ligation reactions. DNA with ligated adaptors was treated with USER Enzyme (NEB) to remove uracil from the adaptor and ‘open’ its hairpin structure, then cleaned using AMPure XP magnetic beads (Beckman Coulter), with beads mixed with DNA in a 0.9:1 ratio. Subsequently, amplification (7 PCR cycles) of the resulting library was performed, during which adaptor sequences were completed and index sequences were incorporated. For indexing, NEBNext Multiplex Oligos for Illumina (Dual Index Primers Set 1) and NEBNext Multiplex Oligos for Illumina (Dual Index Primers Set 2) (NEB) reagent sets were used. Qualitative assessment of the resulting libraries was performed on an Agilent TapeStation 4150 bioanalyzer using High Sensitivity D5000 ScreenTape and High Sensitivity D5000 Reagents (Agilent), and quantitative assessment was performed using real-time PCR with KAPA Library Quantification Kit (KAPA Biosystems) reagents.

The resulting metagenomic libraries were mixed equimolarly into a pool, which was sequenced on an Illumina NovaSeq 6000 instrument at the Genomic Research Resource Center of the Laboratory Complex of the Scientific Center of Genetics and Life Sciences of Sirius University ANO VO. Sequencing was performed in paired-end read mode 151 + 151 bp. Metagenomic library sequencing data were demultiplexed by index sequences introduced during sample preparation using bcl2fastq v2.20.0.422 with default parameters. In total, 76 to 183 million read pairs were obtained for each genomic library.

Primary quality assessment of the obtained deep sequencing data was performed using FastQC v0.11.2. To remove adaptor sequences as well as low-quality sequences, the deep sequencing data obtained on the Illumina NovaSeq 6000 platform were processed with AdapterRemoval v2 (--maxns 0, --trimwindows 1, --trimqualities, --minquality 20, --minadapteroverlap 5, --minlength 101) and fastp (-g, -l 101, -q 20). On average, after filtering, 97.3% of read pairs were retained for further analysis (Supplementary Table S1).

### 4.4. Metagenome Assembly

Metagenomic assembly was performed using SPAdes software (v3.15.4) with the following parameters: metaspades.py --pe1-1 R1.fq.gz --pe1-2 R2_paired.fq.gz -k 21, 33, 55, 77. The minimum contig length accepted for downstream analysis was 500 bp. Assembly characteristics (N50, number of contigs, total assembly size) are presented in Supplementary Table S1.

#### 4.4.1. Gene and MGE Annotation

Gene annotation was performed using Prokka (v1.14.6; [52]) with the --metagenome flag. Antibiotic resistance genes were identified using DeepARG (v2.0; [53]) with a minimum identity threshold of 70% and an e-value cutoff of 1 × 10^−10^ against the DeepARG-DB database (version 2.0). Additional filtering was performed against the UniProt KB database (keyword KW-0046) to retain only sequences with an annotated resistance function. Only ARG predictions with a model probability score ≥ 0.8 were retained for downstream analysis.

Determination of abundance and analysis of nucleotide sequences of 16S rRNA genes in samples were performed using Barrnap (version 0.9) software (https://github.com/tseemann/barrnap, accessed on 5 November 2025) with a set identity threshold of 65% followed by manual filtering [52].

Plasmid sequences were identified using Platon (v1.6; [54]) with default parameters. Contigs with a replicon distribution score (RDS) below 0.6 were classified as plasmid-derived; those with an RDS ≥ 0.6 were classified as chromosomal. All Platon-predicted plasmid candidates were subjected to a BLASTn homology search against the NCBI nucleotide database (e-value < 1 × 10^−5^, identity ≥ 90%, query coverage ≥ 80%) to verify plasmid identity and exclude misclassified chromosomal or other mobile elements. Sequences not matching confirmed plasmid entries were reclassified as non-plasmid MGEs (cosmids, integrons, or chromosomal elements).

For verification of obtained candidates, a homology search in the NCBI database was performed using BLASTn (version 2.17.0). The search for prophage integrase sequences was carried out based on annotations obtained using Prokka (version 1.13.4). Transposase sequences were predicted from translated ORFs (Prodigal v2.6.3, -p meta mode) and classified into families by a profile hidden Markov model (HMM) search using HMMER v3.3.2 (hmmsearch; E-value threshold < 1 × 10^−9^, bit score > 100) against the Pfam transposase domain library (Pfam release 35.0; families: Transposase_mut, Transposase_31, Y1_Tnp, Y2_Tnp, DDE_2, DBD_Tnp_Hermes, DBD_Tnp_Mut).

To identify a physical association between mobile genetic elements and antibiotic resistance genes, their localization (coordinates) on the same contigs at distances not exceeding 10 kb was compared [55].

Resistome load was normalized using the single-copy housekeeping gene *rpoB* (N/*rpoB* ratio) rather than the 16S rRNA gene. This approach was adopted because 16S rRNA genes are present in variable copy numbers (1–15 per genome), which introduces systematic bias in comparative abundance estimates across taxa [38]. The *rpoB* gene is present as a single copy in virtually all bacteria, providing a more accurate denominator for inter-sample normalization of ARG abundance.

#### 4.4.2. Taxonomic Profiling

Taxonomic profiling was performed using Kraken2 (v2.1.3; [56]) against a custom bacterial database (built from NCBI RefSeq bacterial genomes, downloaded 12 October 2025) with a confidence threshold of 0.05. Family-level relative abundances were calculated from classified reads using Bracken (v2.7; min. reads threshold 10). The adjusted Shannon index was used as a diversity measure with a minimum threshold of relative abundance at the family level—0.001% relative to the number of reads classified as bacterial. Analysis of taxonomic composition similarity and β-diversity was conducted using the Bray–Curtis index. Ordination of β-diversity was performed by Principal Coordinates Analysis (PCoA) using the Bray–Curtis dissimilarity matrix as input (cmdscale function, R stats package/sklearn.manifold.MDS). Mg_188 was excluded from ordination owing to anomalously low classification efficiency.

The Kruskal–Wallis test was applied to compare Shannon indices between soil type groups; a non-parametric test was selected given the small and unequal group sizes. The effect size was reported as epsilon-squared (ε^2^). A single planned comparison was performed; no correction for multiple testing was applied. Prior to PERMANOVA, the homogeneity of multivariate dispersions was verified using the betadisper permutation test (999 permutations). For each factor tested in PERMANOVA, R^2^ is reported as a measure of the proportion of variance explained. The four groupings tested represented pre-specified hypotheses; no correction for multiple comparisons was applied.

## 5. Conclusions

This work forms the first metagenomic baseline profile of the resistome and mobilome of West Siberian agroecosystems. The obtained data indicate moderate anthropogenic pressure on soil microbiomes compared to intensively managed agricultural systems in other regions. However, the established mobilization infrastructure, evidenced by a high abundance of class 1 integron integrases (*intI1*/*rpoB* > 1 in 9 of 12 samples) and ARG-MGE associations in 11 of 12 samples, suggests the potential for horizontal gene transfer that exceeds the expected natural background.

Our analysis revealed a stable taxonomic foundation underlying the resistome, with a consistent dominance of *Streptomycetaceae*, *Nitrobacteraceae*, and *Sphingomonadaceae* across all samples regardless of the crop type or soil conditions. This taxonomic stability explains the predominance of efflux genes (*emrA*, *drrA*, *tetA*, *abaF*, *bcr*, *fsr*) and target modification genes (*lnrL*) in the resistome, with normalized loads (N/*rpoB* = 2.30–5.37) reflecting moderate anthropogenic enrichment rather than intensive contamination. The high abundance of mobile genetic elements, with integrases as the primary mobilization vector, indicates conditions favorable for horizontal gene transfer beyond the natural bacterial reservoir.

Of particular note is the detection of *lnrL* in *Pseudomonas* in association with prophage integrase *IntA*, which is consistent with the horizontal transfer of this lincosamide resistance determinant beyond its natural actinomycete reservoir. Additionally, in sample Mg_155, genes *emrA–emrB* and *bcr* (NODE_16) and *arnA* and *lnrL* (NODE_6) were each independently co-localized with distinct prophage *IntA* integrase copies within *Pseudomonas* contigs, documenting multiple parallel mobilization events encompassing resistance to five antibiotic classes. These findings suggest that prophage-mediated transfer may represent an important and currently underappreciated mechanism for ARG dissemination in agricultural soil environments.

The implications for regional antimicrobial resistance surveillance are threefold. First, monitoring programs should expand beyond traditional *intI1* markers to include prophage integrases, which our data identify as equally important mobilization vectors. Second, tracking of clinically relevant genes like *lnrL* in non-actinomycete taxa represents a critical early warning system for resistance spillover from environmental to clinical settings. Third, given that organic fertilizer application regimes represent the most manageable anthropogenic factor influencing ARG loads, agricultural interventions should prioritize pre-treatment of animal waste to reduce both ARG content and mobilization potential. The baseline profile established here provides a foundation for evaluating intervention strategies in this grain-producing region and contributes to the broader understanding of AMR ecology in agricultural landscapes.

## Figures and Tables

**Table 1:** Results of genomic annotation of metagenomic assemblies.

Sample ID	Mg_ 141	Mg_ 142	Mg_ 145	Mg_ 147	Mg_ 155	Mg_ 156	Mg_ 164	Mg_ 173	Mg_ 177	Mg_ 181	Mg_ 184	Mg_ 188	Mean ± SD
Total number of genes (N), n	408	249	820	483	779	418	391	570	689	692	650	309	538 ± 188
Unique genes, n	22	19	24	18	24	22	20	18	25	22	23	49	24 ± 8
Single copy, n	5	2	6	2	6	2	4	3	5	2	2	20	5 ± 5
*rpoB* gene, n	155	108	195	90	165	107	143	147	154	181	187	126	147 ± 34
N/*rpoB*	2.63	2.30	4.21	5.37	4.72	3.91	2.73	3.88	4.47	3.82	3.48	2.45	3.70 ± 1.00

**Table 2:** Functional affiliation of ARGs.

Gene	Resistance Mechanism	Family	Antibiotic Classes
*emrA*, *emrB*	Efflux	MFS (EmrAB-TolC)	Nalidixic acid, novobiocin
*norB*	Efflux	MFS	Fluoroquinolones, chloramphenicol
*drrA*	Efflux	ABC transporter	Anthracyclines
*tetA*	Efflux	MFS	Tetracyclines
*bcr*	Efflux	MFS	Bicyclomycin
*abaF*	Efflux	MFS	Fosfomycin
*fsr*	Efflux	-	Fosmidomycin
*lnrL*	Target modification	Methyltransferase	Oxazolidinones, phenicols
*arnA*	Cell wall modification	L-Ara4N synthesis	Polymyxins (colistin, polymyxin B)

**Table 3:** MGE associations with ARGs on one contig at distances less than 10 kb.

Sample ID	NODE	Position	Gene	Taxonomy	Association		
					Transposase	Integrase	Plasmide
141	34	20,318–20,806	Multiple antibiotic resistance protein *MarR*	*Ralstonia*		*IntS* (30,800–32,014)	
142	34	62,653–62,165	Multiple antibiotic resistance protein *MarR*	*Ralstonia*		*IntS* (50,942–52,150)	
	2,278,419	5222–6677	MFS tetracycline efflux transporter *Tet_64*	*Pseudomonas*		*intI1* (3–299)	
145	5	335,572–336,630	Multidrug efflux membrane fusion protein *EmrA*	*Lacibacter*	Y1_Tnp (344,630–345,081)		
	130	20,318–20,806	Multiple antibiotic resistance protein *MarR*	*Ralstonia*		*IntS* (29,806–31,020)	
155	6	2621–1578	Bifunctional polymyxin resistance protein *ArnA*	*Pseudomonas*		*IntA* (3621–4661)	
	6	212,632–213,558	Bifunctional polymyxin resistance protein *ArnA*	*Pseudomonas*		*IntA* (215,558–215,988)	
	6	271,907–271,020	Linearmycin resistance ATP-binding protein *LnrL*	*Pseudomonas*		*IntA* (281,010–282,050)	
	16	27,256–28,452	Multidrug efflux membrane fusion protein *EmrA*	*Pseudomonas*		*IntA* (31,452–32,492)	
	16	28,458–30,035	Multidrug efflux membrane fusion protein *EmrB*	*Pseudomonas*		*IntA* (31,452–32,492)	
	16	49,150–50,376	Bicyclomycin resistance protein	*Pseudomonas*		*IntA* (53,376–54,416)	
	127	20,318–20,806	Multiple antibiotic resistance protein *MarR*	*Ralstonia*		*IntS* (30,800–32,014)	
	1207	8294–7041	Tetracycline resistance protein, class C	*Aquabacterium*		*IntA* (10,298–11,380)	
156	51	62,653–62,165	Multiple antibiotic resistance protein *MarR*	*Ralstonia*		*IntS* (60,156–61,370)	
164	192	55,380–55,733	Bleomycin resistance protein	*Mucilaginibacter*	Y1_Tnp (56,026–55,814)		
	88	40,700–41,704	Multidrug efflux membrane fusion protein *EmrA*	*Stenotrophomonas*			*Bradyrhizobium* sp. BTAi1
	100	57,076–58,155	Multidrug efflux membrane fusion protein *EmrA*	*Bradyrhizobium*		*IntA* (61,655–62,695)	
	138	20,318–20,806	Multiple antibiotic resistance protein *MarR*	*Ralstonia*		*IntS* (25,806–27,020)	
	45,769	6197–6672	Dihydrofolate reductase type I *dfrA1*	*Proteus*		intI1 (873–1271)	
173	16	107,901–109,076	Bicyclomycin resistance protein	*Burkholderia*		*IntA* (112,676–113,716)	
	13,810	17–1075	Multidrug efflux membrane fusion protein *EmrA*	*Bacteroidota*			*Sphingomonas paucimobilis* strain FDAARGOS_881 plasmid unnamed1
177	16	66,094–67,500	Fosfomycin resistance protein *AbaF*	*Ralstonia*	Transposase_mut (67,602–67,796)		
	51	20,318–20,806	Multiple antibiotic resistance protein *MarR*	*Ralstonia*		*IntS* (28,551–29,765)	
181	1	28,385–29,575	Bicyclomycin resistance protein	*Ralstonia*	Transposase_31 (35,001–35,942)		
	24,211	16–1161	Bicyclomycin resistance protein	*Pseudomonadota*			*Propionibacterium acnes* HL096PA1 plasmid
	76	84,447–85,691	Multidrug efflux membrane fusion protein *EmrA*	*Sphingomonas*			*Bradyrhizobium* sp. BTAi1
	2	53,673–54,752	Multidrug efflux membrane fusion protein *EmrA*	*Bradyrhizobium*	Transposase_31 (58,252–59,152)		
	2	265,246–266,376	Multidrug efflux membrane fusion protein *EmrA*	*Bradyrhizobium*	Transposase_31 (269,876–270,776)		
	2	263,630–265,117	Multidrug efflux membrane fusion protein *EmrB*	*Bradyrhizobium*	Transposase_31 (268,617–269,517)		
	1	885,085–886,326	Fosmidomycin resistance protein	*Ralstonia*	Transposase_31 (881,185–880,265)		
184	10	238,371–239,429	Bifunctional polymyxin resistance protein *ArnA*	*Ralstonia*		*IntA* (230,371–229,331)	
	10	239,446–240,387	Bifunctional polymyxin resistance protein *ArnA*	*Ralstonia*		*IntA* (230,371–229,331)	
	577	202–921	Daunorubicin/doxorubicin resistance ATP-binding protein *DrrA*	*Bradyrhizobium*		*IntA* (8463–9662)	
	21,240	641–1993	Multidrug efflux membrane fusion protein *EmrA*	-	Y1_Tnp (3993–6270)		
188	25	21,827–20,865	Linearmycin resistance ATP-binding protein *LnrL*	-	Transposase_mut (19,827–18,107)		

**Table 4:** Characteristics of collected soil samples.

Sample ID	Coordinates	Soil Type	Cultivated Crop
Mg_141	53.74233, 83.65125	Sandy soils	Sunflower
Mg_142	54.30555, 83.27879	Leached chernozems	Pea
Mg_145	54.69037, 83.20478	Leached chernozems	Wheat
Mg_147	53.95597, 83.43419	Podzolized chernozems	Barley
Mg_155	54.71247, 83.14837	Leached chernozems	Corn
Mg_156	53.99529, 79.22073	Meadow saline and solonetzic soils	Wheat
Mg_164	54.93073, 83.20762	Gray forest soils	Potato
Mg_173	55.27537, 76.51300	Meadow-chernozem saline and solonetzic soils	Wheat
Mg_177	54.11533, 77.61005	Southern tongue chernozems	Wheat
Mg_181	54.35431, 81.80227	Meadow-chernozem soils	Carrot
Mg_184	54.31577, 81.71262	Meadow-chernozem soils	Wheat
Mg_188	54.62449, 83.21882	Leached chernozems	Wheat

## Data Availability

The raw metagenomic sequencing data generated in this study have been deposited in the NCBI Sequence Read Archive (SRA); sample names, SRA accession numbers, and BioProject details are provided in Table S9.

## Supplementary Materials

The following supporting information can be downloaded at: https://www.mdpi.com/article/10.3390/antibiotics15050502/s1, Table S1. Relative abundance (%) of the ten most prevalent bacterial families across metagenomic samples. Table S2. Measures of bacterial community diversity in samples (Shannon index, number of families, Pielou’s evenness index). Table S3. Bray–Curtis distance matrix between metagenomic samples (n = 11, Mg_188 excluded). Table S4. Frequency of occurrence of ARGs (top 10) in metagenomic assemblies. Table S5. Summary table of transposases in metagenomic assemblies (HMMER HMM search). Table S6. Summary table of integrases in metagenomic assemblies (Prokka annotation). Table S7. Summary table of plasmids in metagenomic assemblies (Platon). Table S8. Summary table of ARG–MGE associations in metagenomic assemblies. Table S9: Sequencing statistics and assembly characteristics of metagenomic libraries.
